# Perfuming Sick Rooms

**Published:** 1858-07

**Authors:** 


### PERFUMING SICK ROOMS.

VARIOUS things have been recommended: such as the sprinkling of sugar on burning coals, the odor of roasted coffee, sliced onions, and the like. These things are worse than useless. The odor of the sick-chamber is merely overpowered, it is neither removed nor destroyed; and, by the additional odor of the sugar or coffee, each breath of air becomes more solid, by the displacement of its more yielding, vital qualities—as the same point of space cannot be occupied by a particle of odor and a particle of oxygen at the same time. The odoriferous atom being more material than an atom of oxygen, the latter yields, gives way to the former, so that the expedient is only apparently beneficial; it may be grateful to a visitor, but it is positively hurtful to the invalid. There are some articles which, if dampened with water, absolutely absorb bad odors, such as unslaked lime or pulverized charcoal. Half a pound or less of copperas, dissolved in water, and thrown in a privy, absorbs the odors in a few moments, by its strong attractive affinity for the sulphureted hydrogen. Still, the only safe, certain, and absolutely perfect deodorizer, is a thorough ventilation of the chamber of the sick, and it is a humanity to accomplish it. It should be the study of every nurse and every physician, during every hour of attendance, to promote, in all possible ways, a constant moderate change of atmosphere. This is easily done in fire-time of the year, by keeping the grate or fireplace open, and occasionally opening a window or door opposite. In the summer-time, the most simple and effectual method, is to build a fire of light materials in the fireplace, several times during the day, oftener during the night hours, with the door open all the time; this will inevitably give a gentle circulation, by which sick odors will be driven up the chimney, to be replaced by the fresh out-door air. It is not meant that a fire should be kept burning all the time, of a hot summer's day, but to have a small blaze from very light materials, which will burn out in half an hour. The reason for this is, that in the whole circuit of Nature, the most efficient of all remedial means, in every disease, and without which there can be no perfect recovery, is an abundant and constant supply of a pure, fresh air from without.

